# Perception of COVID-19 Threats among Individuals from Different Countries: A Survey

**DOI:** 10.5152/eurasianjmed.2021.20260

**Published:** 2021-06

**Authors:** Ibrahim Ethem Gurbuz, Hema Sekhar Reddy Rajula, Halil Koca, Vedat Karadeniz, Vassilios Fanos

**Affiliations:** 1 Social Studies Education, Department of Turkish and Social Sciences Education, University of Atatürk, Erzurum, Turkey; 2 Neonatal Intensive Care Unit, Department of Surgical Sciences, AOU and University of Cagliari, Cagliari, Italy; 3 Marie Sklodowska-Curie CAPICE Project, Department of Surgical Sciences, University of Cagliari, Cagliari, Italy; 4 Social Studies Education, Department of Geography Education, University of Atatürk, Erzurum, Turkey; 5 Social Studies Education, Department of Turkish and Social Sciences Education, Faculty of Education, University of Erzincan Binali Yıldırım, Erzincan, Turkey

**Keywords:** pandemic, perception, biological experiment, joint initiative

## Abstract

**Objective:**

The aim is to discover the perceptions of individuals living in different countries relating to Covid-19 and develop a joint initiative against this virus and future outbreaks by making comparisons over a number of sociological factors.

**Materials and Methods:**

A cross-sectional research design was applied, which is a type of descriptive survey belonging to quantitative research. The sample was selected from various countries: Turkey, USA, France, Germany, Netherlands, Georgia, India, and South Africa. The total number of participants is 1020 people. The data were provided through the One-Way Anova Test and collected based on "The Covid-19 Perception Questionnaire" which contained 5 personal information and 10 items.

**Results:**

We found that statistically significant differences among the Covid-19 perceptions of individuals. The effect size showed that this difference is at a large level. As the variances did not evenly distribute, the Dunnett C multiple comparison tests were applied. According to this, the highest mean in Georgia and India, the lowest mean in Germany and the USA.

**Conclusion:**

The majority have the national and international awareness required to cope with the pandemic. However, the source of the virus has still not been explained so it has been observed that the number of people who believe in conspiracy theories is also high. As a result, people need more reliable sources of information, especially the World Health Organization should make more precise explanations to people about the origin of Covid-19 and updated information should be made available to people constantly. In addition, although a long time passed after the appearance of the Covid-19, people are still confused.

## Introduction

History has shown millions of people had died because of various viruses seen in many parts of the world. Viruses are geographically classified as natural disasters of biological origin. Biological natural disasters are events that occur depending on other biological hazards, which cause great damage to human life such as epidemic diseases, insect infestations, and so forth.^1^ The most well-known epidemics caused by the spread of microbes or microorganisms that live on various animals or plants are; Antoninus (Galen) epidemic (AD 165–180), Justinian plague (AD 541), Black plague (1346–1353), chickenpox in American Indians (15^th^ century), Cocoliztli outbreaks (1520–1576), cholera epidemics: Third epidemic (1852–1860), Typhus outbreak (during World War I), Spanish Flu pandemic (1918), Asian Flu pandemic (1957), HIV (AIDS) virus.^2^ Various animals and reptiles were shown as the main cause of all these diseases, and humanity has faced another kind of epidemic that is claimed to have emerged from bats.

According to the World Health Organization (WHO), coronaviruses are a large family of viruses that can cause human and animal diseases, among these respiratory infections such as the Middle East respiratory syndrome (MERS) and severe acute respiratory syndrome (SARS).^3^ They (SARS-CoV and MERS-CoV) lead to severe and potentially fatal respiratory tract infections.^4^ COVID-19, which was recently discovered, is one of them.^3^ Coronavirus was initially named as the 2019-novel coronavirus (2019-nCoV) on 12 January 2020 by WHO.^4^ The SARS-CoV-2 is a β-coronavirus, which is an enveloped, non-segmented, positive-sense RNA virus (subgenus sarbecovirus, Orthocoronavirinae subfamily). They are distributed broadly among humans, other mammals, and birds and cause respiratory, enteric, hepatic, and neurologic diseases.^5^

COVID-19, called the novel type of coronavirus, is thought to have emerged in the Wuhan province of the People’s Republic of China in December 2019.^6^ Although the general opinion reflected in the press is that it appears to have emerged from an animal market which sold mostly seafood, there still remain current discussions that it might have been a biological experiment accident or sabotage.^7^

However, some researchers, in their reports of the COVID-19 situation, describe that SARS-CoV and MERS-CoV that caused outbreaks in the 2000s and 2010s were of zoonotic origin, similar to other coronaviruses. They have stated that SARS-CoV is transmitted from exotic animals and MERS-CoV from camels to people, and both the pathogens are of bat origin. They also stated that some pneumonia cases which were seen on 29 December 2019 were associated with the Huanan Seafood Market.^8^ It appears that most of the early cases had contact history with the original seafood market; however, the disease has now progressed to be transmitted by human-to-human contact.^9^ Thus, it is claimed by scientists that this new virus case is also of animal origin. According to Peiris JS et al.^10^, all information available till date indicates that SARS-CoV is necessary and sufficient for causing SARS in humans; however, it has not yet been determined whether microbial or other cofactors increase the severity of contagiousness of the disease. The exact genetic sequence of the SARS-CoV genome was determined, and it was confirmed to belong to a new group in the coronavirus family.^10^

Extensive research continues into COVID-19 since its emergence; however, if there is a different dimension of the work, can a common consciousness be developed in the face of such a virus that affects all countries of the world? Because for a successful fight against this virus, which has the potential to spread rapidly among people, citizens of all countries of the world must have a certain consciousness. The only way to measure this awareness is to determine the perceptions of individuals against this virus.

Perceptions can vary among individuals, and there is no single truth as it will vary from region to region, country to country; therefore, the facts perceived by individuals may differ.^11^ We conducted this study to clarify some questions that are being asked by the citizens of all the countries, especially the scientists. Accordingly, the following research problem was proposed:

“Does living in a different country have an impact on COVID-19 perception?”

Furthermore, the following hypotheses have been proposed:

H_0_ Hypothesis = Living in a different country has no effect on COVID-19 perception.
H_1_ Hypothesis = Living in a different country has an impact on COVID-19 perception.

In addition, we sought solutions for the following sub-problems:

What are the differences between the perceptions of individuals living in different countries towards COVID-19? What are the similarities between the perceptions of individuals living in different countries towards COVID-19?

In this study, we aimed to determine the perceptions and tendencies of individuals living in different countries on COVID-19 through a number of social facts. We believe our study will help develop a common initiative against both COVID-19 and the different types of outbreaks that may occur in the future.

## Materials and Methods

### Research Design

This research was conducted based on a quantitative approach and cross-sectional research, a type of descriptive survey pattern.^12^ In cross-sectional studies, people with the quality and quantity that can represent the population are chosen by chance, and a result is obtained by getting opinions from them.^12^

Survey studies allow quantitative or numerical definitions can be made by working with a sample selected from this population of the trends, attitudes, or views of the population. In these studies, cross-sectional and longitudinal methods are preferred through structured interviews or data collection questionnaires to generalize the sample to the population.^13^

### Research Sample

The study group was selected from random and volunteer participants from various countries such as Turkey, USA, France, Germany, Netherlands, Georgia, India, and South Africa; and the total number of participants was 1,020.

### Research Instrument and Procedures

COVID-19 Perception Questionnaire Form consists of five personal information and 10 items. The items were prepared in accordance with the 3-point Likert scale option and rated as: Disagree (1), Undecided (2), and Agree (3). A total of 10 items was prepared to use time efficiently and not distract the participants.

There are different methods to estimate the validity and reliability of quantitative research. In this study, the reliability of the measuring tool was made by calculating Cronbach’s alpha reliability coefficient, which is an indicator of how consistent the measurement is in itself, without the need for more than one application.^14^ Before calculating the Cronbach’s alpha reliability coefficient as all the items except the seventh was negative, reverse coding was done, and alpha was found as α = 0.64. The confidence interval for Cronbach’s alpha; the measure consequent is quite reliable if it is between 0.60 ≤ α < 0.90.^14^

To ensure content validity, the measurement tool was presented to three scientists who were experts in the field of Social Sciences and two scientists who were experts in the field of Health Sciences. They were asked to evaluate each item on the basis of three responses as “appropriate,” “should be changed,” or “removed.” The measurement tool was revised according to the feedback received, and no item was required to be removed.

Data were collected using Google Forms (a free online software that allows to create surveys and quizzes). The measurement tool developed by the researchers was translated into English, French, German, Dutch, and Georgian languages through translators; and each one was created separately. The questionnaire form was translated and presented in each country’s language to the participants to provide more reliable and sincere answers. The links related to the measurement tool created with Google.forms tag were provided to the participants on a voluntary basis through social platforms such as “WhatsApp, Facebook, Instagram, Twitter, e-mail link, etc.” The piloting of the questionnaire was administered to a total of 50 participants from Turkey and France.

### Statistical Analysis

Statistical analysis was done through IBM Statistical Package for the Social Sciences version 20 (IBM SPSS Corp.; Armonk, NY, USA). One-way analysis of variance (ANOVA) was used to compare the averages of the participants as there were more than two groups.^15^

Ethics Committee approval for the study was obtained from the Ethic Committee of Ataturk University, Institute of Educational Sciences. (16.07.2020, 29202147-300-E.2000177060.) Informed consent is not required for this study.

## Results

Personal information of the individuals participating in the research from various countries of the world is shown in Table 1.

In descending order, the number of participants for country variable from Turkey, USA, France, India, Germany, Georgia, Netherlands, and South Africa are 23.1% (236), 18.2% (186), 15.2% (155), 12.3% (125), 11.6% (118), 10.2% (104), 8% (82), and 1.4% (14) respectively.

For the sex variable, the number of women (N = 659) was more than men (N = 361).

The ages of the participants were 50.2% (512) between the ages of 18 and 25 years, 27.3% (278) between the ages of 26 and 35 years, 12.8% (131) between the ages of 36 and 45 years, 6.5% between the ages of 46 and 55 years (66), 2.2% (22) between the ages of 56 and 65 years, and 1.1% (11) over the age of 65.

Educational status of the participants were primary school 1.1% (11), secondary school 5.5% (56), high school 11.9% (121), university student or graduate 55.2% (563), master’s degree or graduate 22% (224), and doctoral student or graduate 4.4% (45).

The marital status of the participants was 35.5% (362 participants) married and 64.4% (657) single.

In Table 2,

For item 1, “Coronavirus emerged as a result of a biological experiment,” the responses were 31% (316) “I disagree,” 37.1% (378) “I’m undecided,” and 32% (326) “I agree.”

For item 2, “The news that coronavirus is transmitted from bats is a deception” the responses were 27.2% (277) “I disagree,” 41.9% (427) “I’m undecided,” and 31% (316) “I agree.”

For item 3, “Coronavirus is not as great a danger as it is exaggerated,” the responses were 59.8% (610) “I disagree,” 16.7% (170) “I’m undecided,” and 23.5% (240) “I agree.”

For item 4, “The number of coronavirus cases shown in the news is not real,” the responses were 31.8% (324) “I disagree,” 23.3% (238) “I’m undecided,” and 44.9% (458) “I agree.”

For item 5, “If we eat healthy, coronavirus will not infect us,” the responses were 67.8% (692) “I disagree,” 16.8% (171) “I’m undecided,” and 15.4% (157) “I agree.”

For item 6, “I believe that a vaccine has been found for coronavirus, but it is not released in the interests of the great powers,” the responses were 51.9% (529) “Disagree,” 29.3% (299) “Undecided,” and 18.8% (192) “Agree.”

For item 7, “I think coronavirus will continue for a long time,” the responses were 11.3% (115) “I disagree,” 24.3% (248) “I’m undecided,” and 64.4% (657) “I agree.”

For item 8, “Coronavirus can infect only old people,” the responses were 76.6% (781) “I disagree,” 13.2% (135) “I’m undecided,” and 10.2% (104) “I agree.”

For item 9, “It is unnecessary to have restrictions/curfew because of coronavirus,” the responses were 63.9% (652) “I disagree,” 15.7% (160) “I undecided,” and 20.4% (208) “I agree.”

For item 10 prepared to measure the effect of religion on a social event, “ Coronavirus is a punishment given to people by God,” the responses were 61.6% (628) “I disagree,” 16.9% (172) “I’m undecided,” and 21.6% (220) “I agree.”

**Figure 1:**
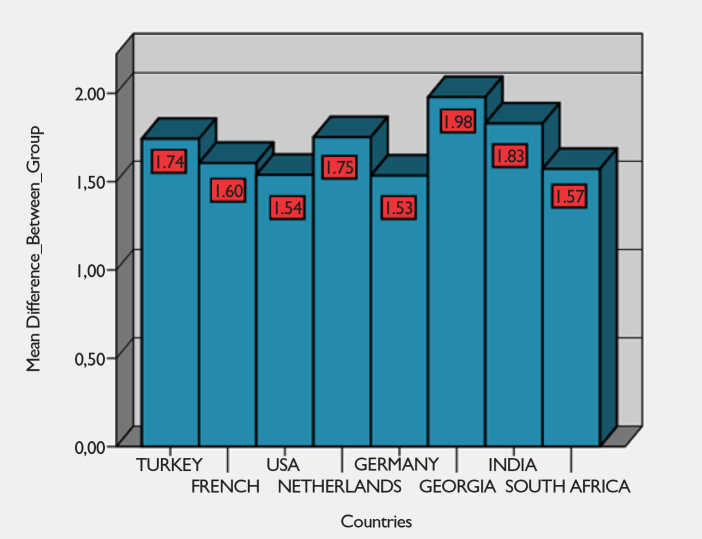
The Means (M) of the Countries

According to Figure 1, means of the countries are close to each other. Moreover, skewness and kurtosis values are found to be between −1.96 and +1.96. These data make us believe that the distribution is normal. In addition, our sample size is large enough, thus we can observe Shapiro-Wilk test for normality.^15^ The result of Shapiro-Wilk tests are (according to α = 0.001 significance level), generally, data distributions are normal, and furthermore graphical (Histogram, Q-Q Plot, Box-Plot, Line Graph) analyzes are examined, and the distributions are tested to be normal in all the countries.^15^ All these data show us that suitable conditions are provided for the analysis of variance (ANOVA) test.

According to Levene’s test (test of homogeneity of variances before ANOVA test), P < .001 was found, and it was concluded that variances were not evenly distributed; and therefore, Dunnett’s C test was applied. According to the results of Dunnett’s C, the statistically significant difference is among Turkey and USA, France, Germany, Georgia; among USA and Turkey, Georgia, India; among France and Turkey, Georgia, India; among Germany and Turkey, Georgia, India; among Georgia and Turkey, USA, France, Germany; among India and USA, France, Germany. According to the responses given to the items, the highest mean is Georgia (M = 1.98) and India (M = 1.83), and the lowest mean is Germany (M = 1.53) and the USA (M = 1.54).

According to this, the means (M) are:

*M_(TURKEY)_* = 1.74, *M_(USA)_* = 1.54, *M_(FRANCE)_* = 1.61, *M_(INDIA)_* = 1.83, *M_(GERMANY)_* = 1.53, *M_(GEORGIA)_* = 1.98, *M_(NETHERLANDS)_* = 1.75, *M_(SOUTH AFRICA)_* = 1.57 and there is a statistically significant difference between the COVID-19 perceptions of individuals living in different countries (*F_(7–1012)_ = 23,93, P < .001*). The effect size showed that this difference is at a large level (*n^2^* = 0.14). According to the responses given to the items, the highest mean was in Georgia and India, and the lowest mean was in Germany and the USA. These results show us that the countries with the most positive (I agree) responses to the items are Georgia and India, whereas the countries with the most negative (I disagree) responses to the items are Germany and the USA. Therefore, we reject the null hypothesis because there is a significant difference between the COVID-19 perceptions of individuals living in different countries.

## Discussion

Whether COVID-19 is a human-made biological experiment or was transmitted from bat and pangolin is still unknown. It is stated in scientific articles and in the reports of the health institutions that the origin of the coronavirus has not been clarified yet, and this virus has emerged from an animal market.^16^ All available evidence for COVID-19 suggests that it has a zoonotic source. Several researchers have been studying the genomic features of COVID-19 and have found that evidence does not support that it was a laboratory construct. A constructed virus would show a mix of known elements within genomic sequences, which is not the case here. However, considering that humans and bats do not live together, it can be said that this disease could be derived from another animal. These animals could be either wild or domestic.^17^ It was found that the genome sequence of COVID-19 is 96.2% identical to a bat CoV RaTG13, whereas it shares 79.5% identity to SARS-CoV. On the basis of virus genome sequencing results and evolutionary analysis, the bat has been suspected as a natural host of the virus, and COVID-19 might be transmitted from bats via unknown intermediate hosts to infect humans. It is clear now that it uses angiotensin-converting enzyme 2 (ACE2), the same receptor as SARS-CoV to infect humans.^4^

Owing to these uncertainties, it is seen as a very probable situation that the participants are undecided for items 1 and 2. For example, in response to whether virus was produced as a biological experiment, the number of participants remaining undecided and the number believing that virus was biologic were very close in Turkey. The majority in India consider the virus to be a biological experiment. In France, we see that the majority are undecided, but the number of those who think that there is no experiment is also high. Majorities in the US and Germany think that they were no experiments. The rates in the Netherlands are very close, and the vast majority in Georgia are undecided.

The president of the WHO, Tedros Adhanom Ghebreyesus, said in his statement on July 7, 2020, that the COVID-19 epidemic continues to accelerate, and it is clear that the epidemic has not yet peaked.^18^ As of the October 05, 2020. data, the number of coronavirus cases in the world has exceeded 34.8 million, and the loss of life from COVID-19 has exceeded 1 million.^17^ These explanations prove that the virus has reached serious dimensions. Certainly, within the scope of combating the virus, it is very pleasing that the vast majority of participants from all the countries responded in parallel to scientific explanations, that is, they were aware that COVID-19 poses a great risk to humanity.

It was observed that the majority of the participants did not believe the number of cases shown in the news; however, it was stated that nobody intentionally misreported cases and deaths, including physicians.^19^

People of all ages can be affected by the virus. The elderly and those with previous health problems (e.g. hypertension, chronic lung and kidney diseases, diabetes, and heart disease) and smokers seem to experience the virus-induced disease more severely.^20^ In addition, according to Lai et al.,^21^ it has been found that the first 15 cases were aged between 25 and 62 years in Korea. Therefore, it was clearly stated that people of all ages can get this disease. Again, it is an extremely important situation within the scope of the study that the vast majority of the participants are aware (conscious) that the virus can be dangerous for all age groups.

The allegations that a vaccine is available but not released to the market owing to the interests of some countries are striking. However, it has not yet been possible to make a clear distinction in scientific articles on this subject. Contrary to the popular belief, the point of hiding a vaccine and the ability of the microbe to be a biological weapon is irrational because inventing a disease, of which 80% is asymptomatic and mostly affects old people, does not bring any economic, administrative, and political gain to any country.^22^ However, there are views defending the opposite of this view also as the effects of COVID-19 has spread worldwide and are accelerating. Although it is obvious that China, where the virus started to spread, has experienced a significant economic contraction in the first quarter of 2020, we are faced with a situation where the growth expectations for the USA have been reduced to around 0.5%.^23^ The epidemic caused closure of factories in China, known as the production workshop of the world. The developments in China, the second-largest economy after the USA, are echoing in the global markets. Food, toys, automotive industry, tourism, cinema, technology, smart devices industry, chip production, aviation, cinema, shopping, and many more products and services depend on China. The production industry in China covers an area that is too large to be filled quickly.^23^

There is no finding yet that people who eat healthy will not get this virus.^24^ Although we cannot establish a direct correlation between healthy eating and COVID-19 (there is no food that can prevent or treat coronavirus transmission alone), it is also a fact that healthy eating increases our body resistance; and thus, our strengthened immunity makes us more protected against viruses.^25^

When the results of similar studies are examined (Kannan et al.^29^; Bhagavathula et al.^28^; Geldsetzer et al.^27^; Pérez-Fuentes et al.^26^; Cori et al.^30^; Malik et al.^31^), it is extremely important for all individuals to act according to accurate and reliable information in the face of a global event to prevent chaos. Determining the perceptions of individuals in such a situation will protect them from turning to wrong attitudes and actions. In particular, the role of global cooperation in combating the epidemic comes to the fore. For example, a society (country) that has the perception that wearing a mask has no effect against the virus can lead to a huge loss of time and savings in combating the virus.^26^–^31^ In addition, it is a sad fact that during the pandemic process, all countries abide by false information on social media, and even scientific articles published have to be reviewed.^32^

The important goals of this study were to determine the possible wrong perceptions and attitudes (conspiracy theories) of the citizens of various countries in the fight against a global virus, reveal these perceptions, and provide important evidence regarding intended social perceptions to global health organizations.

In conclusion, as a result of the data obtained from this study, we determined that the COVID-19 perceptions in different countries vary from each other according to some items. However, when countries are evaluated in general, there are some ambiguities regarding the source of the cause that caused the virus. Thus, some countries think that the virus was a biological experiment, whereas some countries think that this virus was of animal origin (bat) and are undecided. However, the vast majority in all countries are aware that the virus is a serious danger. In addition, it was observed that the participants did not have confidence in the number of cases shown on the news. In the context of combating the virus, it is important that the vast majority of respondents know that the virus poses a risk to all age groups as a contrary belief could cause this rapidly spreading virus to take many more lives. Unfortunately, again, most of the participants believed that the virus would continue for a long time. This sign of hopelessness can negatively affect the course of the fight against the virus. To prevent this, it would be beneficial if international health organizations shared continuous developments. It was striking that the majority of all participants wanted restrictions to be imposed if necessary. This finding should be considered by the governments of the countries and applied as a measure to prevent the spread of the virus. Finally, it was observed that the negative effect of the virus did not vary on the basis of different religious structures present in the countries, that is, there was no preventive effect of having different beliefs in combating the virus.

**Main Points**

- There are differences in the perspectives of individuals living in different countries according to the items relating to COVID-19.
- According to the vast majority of respondents from different countries, curfew restrictions can be applied as a precaution to reduce the transmission rate of the virus.
- People should be more informed, especially by international health organizations, and updated information should be made available to people constantly.
- Although people have different religious structures, it can be said that they show a common approach to the virus.
- Global organizations need to be in great cooperation. Thus, people can access to real and reliable information instead of believing in conspiracy theories.

## Figures and Tables

**Table 1:** Findings Relating to Personal Variables

	Variables	Frequency (f)	Percent (%)
Country	Turkey	236	23,1
	The USA	186	18,2
	France	155	15,2
	India	125	12,3
	Germany	118	11,6
	Georgia	104	10,2
	The Netherlands	82	8
	South Africa	14	1,4
Sex	Female	659	64,6
	Male	361	35,4
Age (years)	18–25	512	50,2
	26–35	278	27,3
	36–45	131	12,8
	46–55	66	6,5
	56–65	22	2,2
	65+	11	1,1
Educational Status	Primary school	11	1,1
	Middle school	56	5,5
	High school	121	11,9
	University student or graduate	563	55,2
	Master’s degree or graduate	224	22
	Doctoral student or graduate	45	4,4
Marital Status	Married	362	35,5
	Single	657	64,4

**Table 2:** Findings Relating to Items

Countries	Responses	Item 1	Item 2	Item 3	Item 4	Item 5	Item 6	Item 7	Item 8	Item 9	Item 10
TURKEY	Disagree	30	35	169	79	140	84	22	184	206	117
	Undecided	98	88	33	59	46	84	49	36	13	44
	Agree	108	113	34	98	50	68	165	16	17	75
USA	Disagree	80	63	114	75	160	119	20	159	115	157
	Undecided	69	83	29	43	21	34	44	15	27	26
	Agree	37	40	43	68	5	33	122	12	44	3
FRANCE	Disagree	61	47	85	29	139	108	14	142	74	126
	Undecided	69	69	32	33	12	32	60	9	39	23
	Agree	25	39	38	93	4	15	81	4	42	6
INDIA	Disagree	26	35	78	48	56	61	11	59	77	40
	Undecided	38	49	20	21	27	46	18	37	27	29
	Agree	61	41	27	56	42	18	96	29	21	56
GERMANY	Disagree	66	46	73	53	81	82	10	107	70	96
	Undecided	30	40	16	22	17	26	22	6	21	10
	Agree	22	32	29	43	20	10	86	5	27	12
GEORGIA	Disagree	16	18	35	19	54	22	26	62	62	23
	Undecided	50	66	23	40	29	56	41	20	12	30
	Agree	38	20	46	45	21	26	37	22	30	51
NETHERLANDS	Disagree	30	26	47	14	52	45	9	55	38	59
	Undecided	23	28	14	19	15	19	12	11	20	9
	Agree	29	28	21	49	15	18	61	16	24	14
SOUTH AFRICA	Disagree	7	7	9	7	10	8	3	13	10	10
	Undecided	1	4	3	1	4	2	2	1	1	1
	Agree	6	3	2	6	-	4	9	-	3	3

## References

[1] Sahin C, Sipahioglu S (2002). Doğal Afetler ve Türkiye.

[2] Aktan S (2020). Why did the deadliest epidemics in history appear and how did they end?. Euronews.

[3] World Health Organization (2020). Q & A on coronaviruses (COVID-19).

[4] Guo YR, Cao QD, Hong ZS (2020). The origin, transmission and clinical therapies on coronavirus disease 2019 (COVID-19) outbreak – an update on the status. Mil Med Res.

[5] Zhu N, Zhang D, Wang W (2020). A novel coronavirus from patients with pneumonia in China. N Engl J Med.

[6] Republic of Turkey Ministry of Health (2020). New coronavirus disease (COVID-19).

[7] Nesvetailova A, Palan R (2020). Sabotage and the Covid-19 crisis. The Mint Magazine.

[8] Er AG, Unal S (2020). Dünyada ve Türkiye’de 2019 koronavirüs pandemisi. FLORA.

[9] Zhou P, Yang XL, Wang XG (2020). A pneumonia outbreak associated with a new coronavirus of probable bat origin. Nature.

[10] Peiris JS, Yuen KY, Osterhaus AD, Stöhr K (2003). The severe acute respiratory syndrome. N Engl J Med.

[11] Bakan I, Kefe I (2012). Kurumsal açıdan algı ve algı yönetimi. Kahramanmaraş Sütçü İmam Üniv İktis ve İdari Bilim Fak Derg.

[12] Sonmez V, Alacapinar FG (2018). Örneklendirilmiş bilimsel araştırma yöntemleri.

[13] Creswell JW, Demir SB (2017). Research Design: Qualitative, Quantitative and Mixed Methods approaches.

[14] Can A (2017). SPSS ile Bilimsel Araştırma Sürecinde Nicel Veri Analizi.

[15] Pallant J, Balci S, Ahi B (2017). SPSS survival manual: a step by step guide to data analysis using IBM SPSS.

[16] Republic of Turkey Ministry of Health (2020). Covid-19 (SARS-CoV-2 Enfeksiyonu) rehberi.

[17] World Healthy Organization (2020). Coronavirus disease 2019 (COVID-19) situation report 196.

[18] BBC News (2020). Coronavirus map: the number of coronavirus cases in the world has exceeded 37 million what is the latest situation in the countries?.

[19] Sener N (2020). Are the death numbers hidden?. Hurriyet Newspaper.

[20] UNICEF Turkey (2020). Fact or legend? What do you know about coronavirus (COVID-19)?.

[21] Lai C, Liu YH, Wang C (2020). Asymptomatic carrier state, acuterespiratory disease, and pneumonia due to severe acute respiratory syndrome coronavirus 2 (SARS-CoV-2): facts and myths. J Microbiol Immunol Infect.

[22] Grubu Bayindir Saglik (2020). COVID-19 myths and facts! What’s true and what’s false about COVID-19.

[23] Bal E (2020). Coronavirus how will affect the world economy and Turkey?. Evrensel.

[24] Yeditepe University (2020). Coronavirus (Covid-19) nutrition guide.

[25] Turkish Dietetic Association COVID-19 beslenme önerileri.

[26] Pérez-Fuentes MDC, Molero Jurado MDM, Oropesa Ruiz NF (2020). Questionnaire on perception of threat from COVID-19. J Clin Med.

[27] Geldsetzer P (2020). Use of rapid online surveys to assess people’s perceptions during infectious disease outbreaks: a cross-sectional survey on COVID-19. J Med Internet Res.

[28] Bhagavathula AS, Aldhaleei WA, Rahmani J, Mahabadi MA, Bandari DK (2020). Knowledge and perceptions of COVID-19 among health care workers: cross-sectional study. JMIR Public Heal Surveill.

[29] Kannan S, Shaik Syed Ali P, Sheeza A, Hemalatha K (2020). Covid-19 (novel coronavirus 2019) - recent trends. Eur Rev Med Pharmacol Sci.

[30] Cori L, Bianchi F, Cadum E, Anthonj C (2020). Risk perception and COVID-19. Int J Environ Res Public Health.

[31] Malik YS, Kumar N, Sircar S (2020). Coronavirus disease pandemic (COVID-19): challenges and a global perspective. Pathogens.

[32] Gupta L, Gasparyan AY, Misra DP, Agarwal V, Zimba O, Yessirkepov M (2020). Information and misinformation on COVID-19: a cross-sectional survey study. J Korean Med Sci.

